# Tracking Dengue Virus Intra-host Genetic Diversity during Human-to-Mosquito Transmission

**DOI:** 10.1371/journal.pntd.0004052

**Published:** 2015-09-01

**Authors:** Shuzhen Sim, Pauline P. K. Aw, Andreas Wilm, Garrett Teoh, Kien Duong Thi Hue, Nguyet Minh Nguyen, Niranjan Nagarajan, Cameron P. Simmons, Martin L. Hibberd

**Affiliations:** 1 Infectious Diseases, Genome Institute of Singapore, Singapore, Singapore; 2 Oxford University Clinical Research Unit, Hospital for Tropical Diseases, Ho Chi Minh City, Vietnam; 3 Centre for Tropical Medicine, Nuffield Department of Clinical Medicine, University of Oxford, Oxford, United Kingdom; 4 Department of Microbiology and Immunology, University of Melbourne, Carlton, Victoria, Australia; Fundaçao Oswaldo Cruz, BRAZIL

## Abstract

Dengue virus (DENV) infection of an individual human or mosquito host produces a dynamic population of closely-related sequences. This intra-host genetic diversity is thought to offer an advantage for arboviruses to adapt as they cycle between two very different host species, but it remains poorly characterized. To track changes in viral intra-host genetic diversity during horizontal transmission, we infected *Aedes aegypti* mosquitoes by allowing them to feed on DENV2-infected patients. We then performed whole-genome deep-sequencing of human- and matched mosquito-derived DENV samples on the Illumina platform and used a sensitive variant-caller to detect single nucleotide variants (SNVs) within each sample. >90% of SNVs were lost upon transition from human to mosquito, as well as from mosquito abdomen to salivary glands. Levels of viral diversity were maintained, however, by the regeneration of new SNVs at each stage of transmission. We further show that SNVs maintained across transmission stages were transmitted as a unit of two at maximum, suggesting the presence of numerous variant genomes carrying only one or two SNVs each. We also present evidence for differences in selection pressures between human and mosquito hosts, particularly on the structural and NS1 genes. This analysis provides insights into how population drops during transmission shape RNA virus genetic diversity, has direct implications for virus evolution, and illustrates the value of high-coverage, whole-genome next-generation sequencing for understanding viral intra-host genetic diversity.

## Author Summary

Dengue virus (DENV) is transmitted between humans through the bite of infected female *Aedes aegypti* mosquitoes. Virus populations experience significant drops in size and are subject to differing selection pressures as they cycle between human and mosquito hosts. Subsequent changes in viral intra-host genetic diversity may have consequences for the adaptability and fitness of the virus population as a whole but are poorly understood. To study the impact of human-to-mosquito transmission on DENV populations, we allowed mosquitoes to feed directly on patients with acute dengue infections, then deep-sequenced DENV populations from patient plasma samples and from the abdomens and salivary glands of corresponding mosquitoes. These matched samples allowed us to estimate the size of the population drop that occurs during establishment of infection in the mosquito, track changes in viral intra-host variant repertoires at different stages in transmission, and investigate the possibility of host-specific immune selection pressures acting on the virus population. These novel insights improve our understanding of DENV population dynamics during horizontal transmission.

## Introduction

With 3.6 billion people at risk and nearly 400 million infections annually [1,2], dengue has become the most important mosquito-borne viral disease affecting humans today. Dengue virus (DENV) is a positive-sense, single-stranded RNA virus of the family *Flaviviridae*, genus *Flavivirus*. The ~10.7 kb DENV genome encodes three structural proteins (capsid [C], premembrane [prM], and envelope [E]) and seven non-structural (NS) proteins (NS1, NS2A, NS2B, NS3, NS4A, NS4B, and NS5).

DENV is transmitted between humans by the mosquitoes *Aedes aegypti* and *Aedes albopictus*. which acquire the virus by taking a bloodmeal from an infected human. Once ingested, DENV first infects and replicates in the mosquito midgut epithelium. It subsequently disseminates through the hemolymph to infect other organs such as the fat body and trachea, finally reaching the salivary glands, where it is secreted into mosquito saliva and injected into a human host during a subsequent blood-feeding event [3].

As is typically the case for RNA viruses, the DENV RNA-dependent RNA polymerase (RdRp, encoded by DENV NS5) operates at low fidelity, resulting in the accumulation of a population of closely-related but genetically-distinct viral genomes, organized around a consensus sequence, within each individual human or mosquito host. Sometimes termed a quasispecies, these intra-host variants are thought to interact cooperatively on a functional level, and to collectively contribute to the overall fitness of the virus population (reviewed in [4]). This has important implications for viral pathogenesis: High fidelity poliovirus mutants, for example, are highly attenuated, demonstrating the importance of genetic diversity and the ability to adapt to changing environments during infection [5,6].

Adaptability is especially important for mosquito-borne viruses, which encounter distinct selection pressures when cycling between vertebrate and invertebrate hosts. Even within a single host, intra-host variants generated through multiple rounds of virus replication are subject to evolutionary mechanisms such as bottlenecks, drift, and positive and negative selection pressures. In human-derived DENV populations, highly immunogenic E protein domains also display higher levels of intra-host genetic diversity, suggesting that selection pressures on low frequency population variants operate even during acute infection [7]. In mosquitoes, RNA interference (RNAi), a key antiviral defense mechanism in insects, has been proposed to be a driver of viral intra-host genetic diversity in the *Culex-*West Nile virus (WNV, family *Flaviviridae*) system [8], where higher intra-host diversity levels have been reported in the mosquito than in vertebrate hosts [9,10].

Host alternation also subjects arboviruses to frequent and significant drops in population size. Within the mosquito and vertebrate hosts, these drops occur during initial establishment of infection and subsequent spread through various tissues and organs, as well as during the process of blood-feeding itself, where only a small percentage of the total virus population circulating in the human actually seeds infection in the mosquito host. It is unclear how such drops shape the diversity and repertoires of intra-host virus populations, and the potential effect they have on transmission.

Studies on intra-host genetic diversity in DENV have focused on virus populations in the human, and with few exceptions [7], have been confined to the sequencing of one or two viral genes [11–14]. It is unclear, however, what happens to intra-host diversity during replication in the mosquito, and particularly after the virus population has gone through a significant drop, such as occurs when a mosquito feeds on a dengue patient that has a viremia around the 50% mosquito infectious dose (MID50).

In this study, we used whole-genome amplification and next-generation Illumina sequencing to characterize DENV intra-host genetic diversity in both patient- and matched mosquito-derived virus populations. Because mosquitoes in our study were infected by allowing them to feed directly on patients, we were able to track changes in viral populations during human-to-mosquito transmission. We used a rigorous variant-calling algorithm to identify low frequency single nucleotide variants (SNVs) within a viral population. This unique study design provides important insights into how population drops shape viral genetic diversity, as well as into the differing selection pressures between human and insect hosts.

## Methods

### Ethics statement

Study protocols were reviewed and approved by the Scientific and Ethical Committee of the Hospital for Tropical Diseases (CS/ND/09/24) and the Oxford Tropical Research Ethical Committee (OxTREC 20–09). Written informed consent was obtained from all participants or from the parent or guardian of any child participants on their behalf.

### Experimental exposure of patients to *Aedes aegypti* mosquitoes

The human viremic plasma and DENV2-infected mosquitoes used here originated from the study by Nguyen *et al*. [15], in which field-derived *Ae*. *aegypti* mosquitoes were allowed to feed on acute dengue cases. Just prior to blood feeding, venous blood was drawn and the plasma fraction stored. Blood-fed mosquitoes were reared until day 14 when the abdomen and salivary glands were dissected and stored.

Out of the 83 DENV2-infected patients described by Nguyen *et al*. [15], we selected 12 patient plasma samples and 36 paired blood fed mosquitoes (three per patient) for investigation of DENV sequence diversity in this study. The rationale for these patient-mosquito pairs was that six of them involved mosquito cohorts that blood fed when the plasma viremia was near to the calculated MID50 (S1 Fig) [15]. Hence the seeding of initial infection in these mosquitoes was most likely by a small or very small population of infectious virions, i.e. a major drop in DENV population size moving from human blood to mosquito. Nucleic acid was extracted from plasma samples and from mosquito tissues for quantification of the DENV RNA concentration as described previously [15].

### Whole-genome amplification and sequencing

Next-generation whole-genome sequencing of human- and mosquito-derived DENV samples was performed as described in [16]. Viral RNA was extracted from human plasma or mosquito abdomen / salivary gland samples using the QIAamp Viral RNA Mini Kit (Qiagen), and cDNA synthesis was carried out with the Maxima H Minus First Strand cDNA Synthesis Kit (ThermoScientific) using a single primer designed to bind to the 3' end of the viral genome. The entire DENV genome was PCR-amplified in 14 overlapping fragments, each about 2kb in length, using the PfuUltra II Fusion HS DNA Polymerase (Agilent Technologies). Primer sequences can be found in S5 Table. PCR products were run on an agarose gel and purified using the Qiagen Gel Extraction Kit (Qiagen).

For each sample, equal amounts of all PCR-amplified fragments were combined and sheared on the Covaris S2 sonicator (Covaris) to achieve a peak size range of 100–300 bp (shearing conditions: duty cycle—10%; intensity—5; cycles per burst—200; time—110 seconds). Samples were purified with the Qiagen PCR Purification Kit (Qiagen) and quality-checked on the Agilent 2100 Bioanalyzer with a DNA 1000 Chip (Agilent Technologies).

Library preparation was performed with the KAPA Library Preparation Kit (KAPA Biosciences). After end-repair, A-tailing, and adapter ligation, ligated products in the 200–400 bp range were gel-extracted with the Qiagen Gel Extraction Kit (Qiagen). Samples were subjected to 14 PCR cycles to incorporate multiplexing indices, quantified with the KAPA SYBR FAST qPCR Master Mix (KAPA Biosciences) on the LightCycler 480 II real-time thermocycler (Roche Applied Science), and pooled.

Paired-end, multiplexed sequencing (2 x 101 bp reads) of libraries was performed on the Illumina HiSeq (Illumina) at the Genome Institute of Singapore. Base calling was done with CASAVA 1.7; reads that did not pass Illumina’s chastity filter (CASAVA 1.7 user guide) were removed.

### Mapping and Single Nucleotide Variant (SNV) calling

Illumina-generated FASTQ files were put through the Viral Pipeline Runner (ViPR, available at https://github.com/CSB5/vipr), which automates the following steps: Iterative mapping of reads against the DENV-2 reference sequence NC_001474 using the Burrows-Wheeler Aligner [17], generation of a consensus sequence, and calling of low frequency single nucleotide variants (SNVs) against the consensus using the LoFreq algorithm [18]. SNVs differ from single nucleotide polymorphisms (SNPs) in that the latter occur between the consensus sequences of different individuals.

LoFreq has previously been applied to DENV datasets, and its SNV predictions on these datasets have been experimentally validated down to 0.5% frequency [18]. To increase specificity and keep only conservative predictions, SNVs that fulfilled any of the following criteria were discarded: located within primer sequences, located adjacent to known homopolymer regions, coverage of <1000X, frequency of <0.01 (1%).

### Classification and Illumina sequencing of clinical samples from the Early DENgue study (EDEN)

Patient DENV clinical samples from the Early DENgue study (EDEN) [19] were previously assigned into human-human transmission pairs thought to be separated by a single mosquito [20]. This was based on Sanger sequence similarity, distance between physical addresses, and time between dates of fever onset [20]; the spatiotemporal criteria are consistent with the National Environment Agency's criteria for active transmission [21]. Whole-genome Illumina sequencing and SNV calling for these samples was carried out as described above.

### Identifying mutational hot- and coldspots

To identify mutational hotspots, a scanning window approach was used to scan the DENV genome (window size of 20, overlap of five nucleotides) for an excess of SNVs in a window compared with the genome-wide average (binomial test; Bonferroni-corrected p-value < 0.05). This was done on a per-sample basis. For coldspots, SNVs from all samples were pooled and scanned for windows (minimum size of 40) with a depletion of SNVs (binomial test; Bonferroni-corrected p-value < 0.05) [18].

### Clustering SNVs into viral genomes

Since haplotype reconstruction is a notoriously difficult problem, we resorted to a simple approach that estimates a lower bound of the number of haplotypes (viral genomes) present in a sample. This was done by greedily clustering SNVs based on their allele frequency confidence intervals (Agresti-Coull at the 0.05 level). SNVs were sorted by their allele frequency and the SNV with highest allele frequency seeds the first cluster. Variants were added to an existing cluster if their upper confidence interval limit was greater than the cluster minimum, otherwise they form a new cluster. The number of clusters represents a lower bound of the number of distinct viral genomes present in a sample. This clustering approach is implemented as one of the tools that come bundled with LoFreq [18].

### Estimating virus population drops during transmission

The size of the infecting virus population was estimated by simulating 1000 samplings of varying size from the virus population in the human, following a normal distribution with mean SNV frequency matching that in the human. This sampling was carried out for a range of mean SNV frequencies. The sampling error rate was calculated from the simulated SNV frequencies, and p-values were computed using a two-tailed t-distribution (S3 Table).

## Results

### Human-to-mosquito DENV2 transmission

Nguyen *et al*. [15] infected field-derived *Ae*. *aegypti* mosquitoes with DENV2 by allowing them to blood-feed directly on patients at the acute stage of infection. To track changes in viral intra-population genetic diversity during human-to-mosquito transmission, we performed whole-genome Illumina sequencing of DENV2 populations from 12 patient plasma samples and 36 infected mosquitoes (three per patient) derived from that study [15]. We successfully obtained genome-length DENV2 sequences from all 12 patient plasma samples, as well as from 25 mosquitoes (21 abdomen-derived and 25 salivary-gland-derived). We were unable to PCR-amplify virus from the remaining 11 mosquito samples. Successfully sequenced patient and mosquito samples are described in S1 Table.

Single nucleotide variants (SNVs) in each DENV2 population were called with the LoFreq variant calling algorithm [18]. At a conservative minimum cutoff of 1% frequency, we observed a total of 1,116 SNVs across all human- and mosquito-derived sequence sets. These were evenly distributed across the ~10.7 kb DENV2 genome (S2 Fig).

### Virus replication in human and mosquito hosts yields similar levels of intra-host diversity

**Fig 1:**
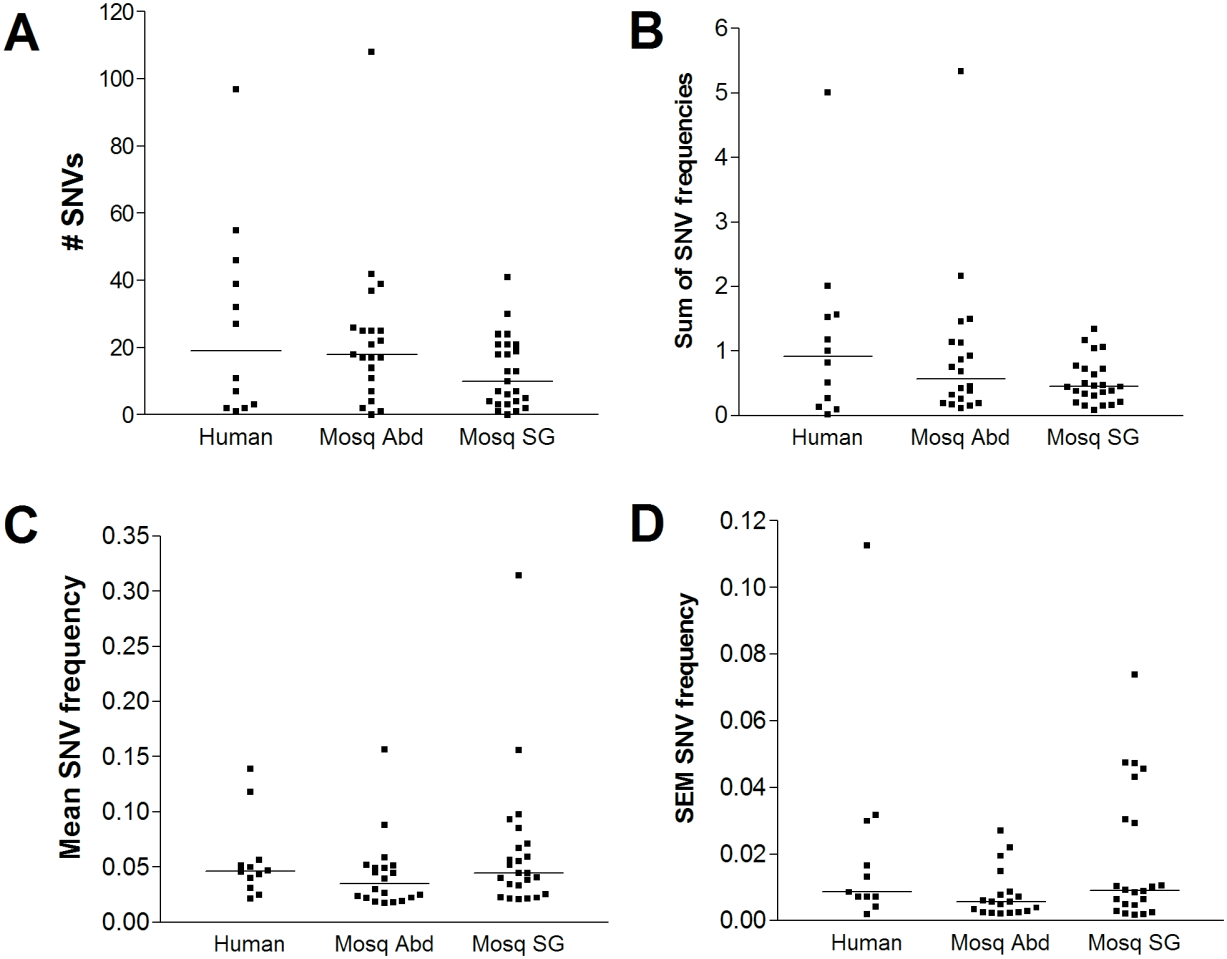
Measures of intra-host DENV2 diversity in human and mosquito hosts. **(A)** Number of SNVs; **(B)** Sum of SNV frequencies; **(C)** Average SNV frequency; **(D)** Standard error of the mean SNV frequency; all calculated on a per sample basis. Abd, abdomen; SG, salivary gland.

We examined four measures of intra-sample diversity, calculated on a per sample basis [7]: a) the number of SNVs, b) the sum of SNV frequencies, c) the average SNV frequency, and d) the standard error of the mean (SEM) SNV frequency (Fig 1). These measures capture different aspects of diversity: the number of SNVs indicates how many distinct variant positions there are along the viral genome; the sum and mean of SNV frequencies indicate how commonly these variants occur and whether they are distributed across many or a few positions; the SEM indicates whether sample diversity comes from dominant variant genomes, minor variant genomes, or a mix of both.

All four diversity measures varied widely among individual samples (Fig 1A–1D). No statistically significant differences in any of these four measures were observed between DENV populations from human plasma, mosquito abdomens and mosquito salivary glands (p > 0.05, Kruskal-Wallis test) (Fig 1A–1D), indicating that intra-sample diversity levels remain similar during replication in human and mosquito hosts.

Although we cannot experimentally follow transmission of DENV2 from mosquitoes into humans, we had access to patient plasma samples from the Early DENgue (EDEN) study carried out in Singapore from 2005–2007 [19]. DENV1 and DENV3 samples from this study were previously assigned to human-human transmission pairs thought to be separated by a single mosquito, based on Sanger sequence similarity and spatiotemporal relationships (distance between patients' homes and time between dates of fever onset) between members of a pair [20]. Illumina deep sequencing of these samples revealed that measures of intra-sample diversity also did not change significantly between members of a human-human transmission pair (S3A–S3D Fig), suggesting that passage through a mosquito does not affect intra-host viral diversity levels upon subsequent replication in the human.

### SNV repertoires change dramatically during human-to-mosquito transmission

**Fig 2:**
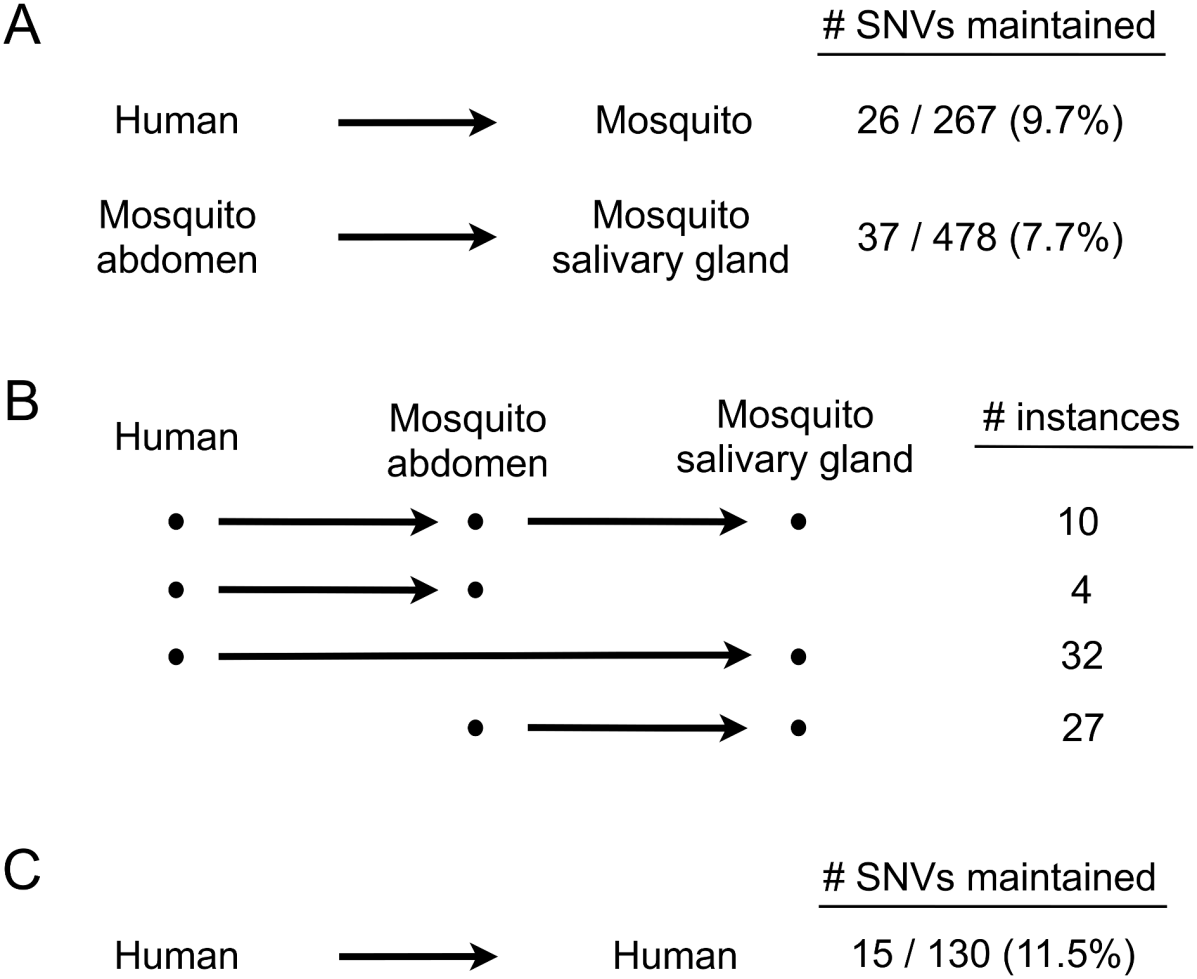
Loss and maintenance of SNVs during horizontal transmission. **(A)** Percentage of SNVs maintained during transmission from human to mosquito and from mosquito abdomen to mosquito salivary gland. **(B)** Tracking of maintained SNVs. The number of instances of each scenario are shown; these include all occurrences of SNVs that were found to be maintained in more than one mosquito. **(C)** Percentage of SNVs maintained between members of a human-human transmission pair from the EDEN study.

Although levels of intra-host diversity remain unchanged, horizontal transmission may alter the SNV repertoire in a viral population if existing SNVs are lost and new ones generated. To examine this, we tracked individual SNVs during transmission from human to mosquito abdomen to mosquito salivary glands (Fig 2). Of 267 SNVs present in human plasma-derived DENV2 populations, only 26 (9.7%) were observed again in any of the associated mosquitoes (abdomen, salivary glands, or both), and only 37 of 478 SNVs present in the mosquito abdomen (7.7%) were observed again in the salivary gland (Fig 2A). This indicates that viral populations are able to quickly restore their diversity upon replication in a new compartment, but predominantly with a very different SNV repertoire that most likely arises through random mutation. It is possible that SNVs not observed in the mosquito abdomen or salivary gland had dropped below our conservatively-chosen level of detection (frequency < 1%).

**Fig 3:**
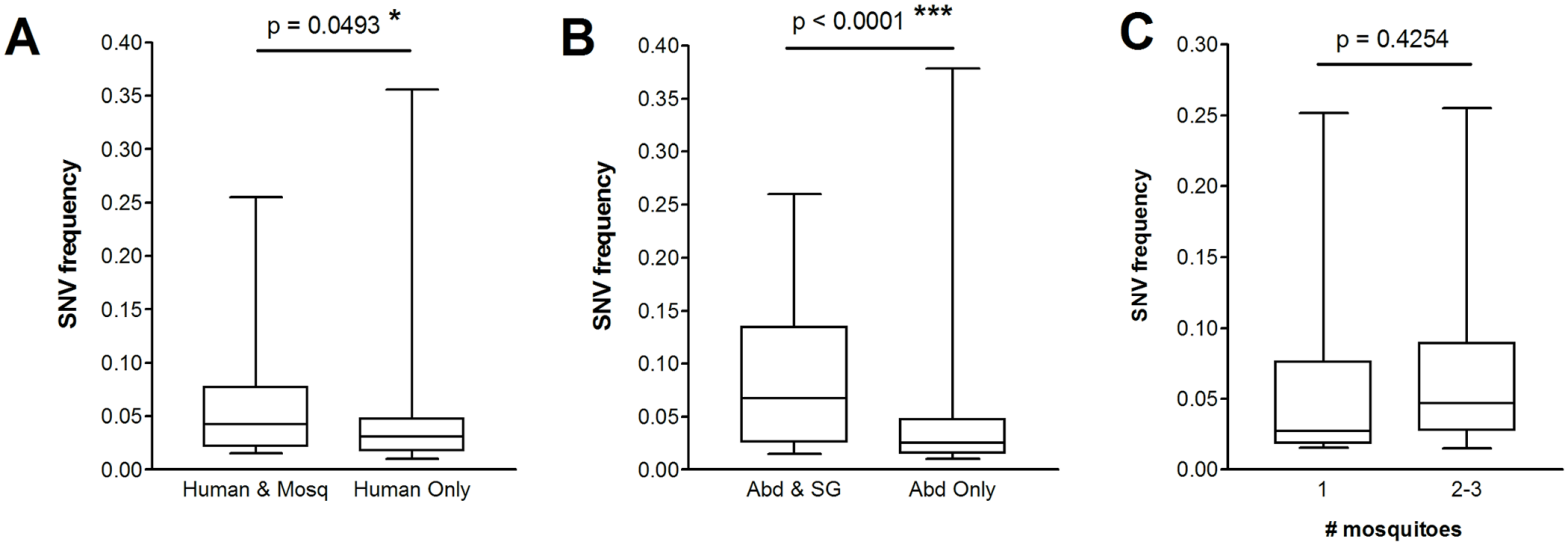
Frequencies of maintained SNVs compared to SNVs lost during horizontal transmission. **(A)** Frequencies of DENV2 SNVs maintained from human to mosquito, versus those present only in the human. **(B)** Frequencies of DENV2 SNVs maintained from mosquito abdomen to mosquito salivary gland, versus those present only in the abdomen. **(C)** Frequencies of SNVs maintained in one mosquito per human-mosquito group, versus those maintained in more than 1 mosquito per group. Boxplots indicate medians and 25th and 75th percentiles; whiskers indicate minimum and maximum values. *, p < 0.05; ***, p < 0.0001; Mann-Whitney test.

We observed relatively few instances of SNVs being maintained across transmission stages (Fig 2B). These SNVs were present at a significantly higher frequency than those that were lost (Fig 3A and 3B), suggesting that higher frequency SNVs are more likely to be detectably transmitted. Maintained SNVs were never seen in more than one human-mosquito group, but were observed in more than one mosquito per human approximately half the time (12 out of 26 cases; in 8 of these cases, SNVs were maintained in all three mosquitoes that had fed on the same human) (Table 1). No significant difference in frequency was observed between SNVs maintained in one mosquito and SNVs maintained in more than one mosquito (Fig 3C).

**Table 1:** SNVs maintained from human to mosquito.

Position	#Hu / Mosq groups	# Mosq / group	Gene	SNV	NS or S	From (aa)	To (aa)
131	1	2	C	C>T	NS	P	L
507	1	3	prM	T>C	S	L	L
1745	1	3	E	T>C	NS	I	T
2457	1	1	NS1	A>G	S	E	E
3114	1	1	NS1	C>T	S	L	L
3594	1	1	NS2A	T>C	S	S	S
3621	1	1	NS2A	G>T	NS	M	I
3949	1	1	NS2A	G>A	NS	A	T
3989	1	2	NS2A	T>C	NS	I	T
4710	1	3	NS3	G>A	S	K	K
5910	1	3	NS3	A>G	S	R	R
6363	1	2	NS3	A>G	S	A	A
7060	1	1	NS4B	G>A	NS	G	G
7410	1	3	NS4B	C>T	S	T	T
7857	1	1	NS5	T>C	S	N	N
8139	1	1	NS5	T>C	S	V	V
8783	1	1	NS5	G>A	NS	S	N
9247	1	2	NS5	G>A	NS	E	K
9384	1	1	NS5	T>C	S	G	G
9596	1	3	NS5	A>G	NS	N	S
9630	1	1	NS5	C>T	S	I	I
9648	1	1	NS5	A>G	S	Q	Q
9705	1	3	NS5	T>C	S	H	H
9837	1	1	NS5	G>A (hu) / G>C (mosq)	S (hu) / NS (mosq)	K	K/N
9876	1	1	NS5	G>A	S	R	R
9968	1	3	NS5	A>G	NS	K	R

NS, non-synonymous; S, synonymous; aa, amino acid.

### Frequencies of maintained SNVs remain similar during human-to-mosquito transmission

**Fig 4:**
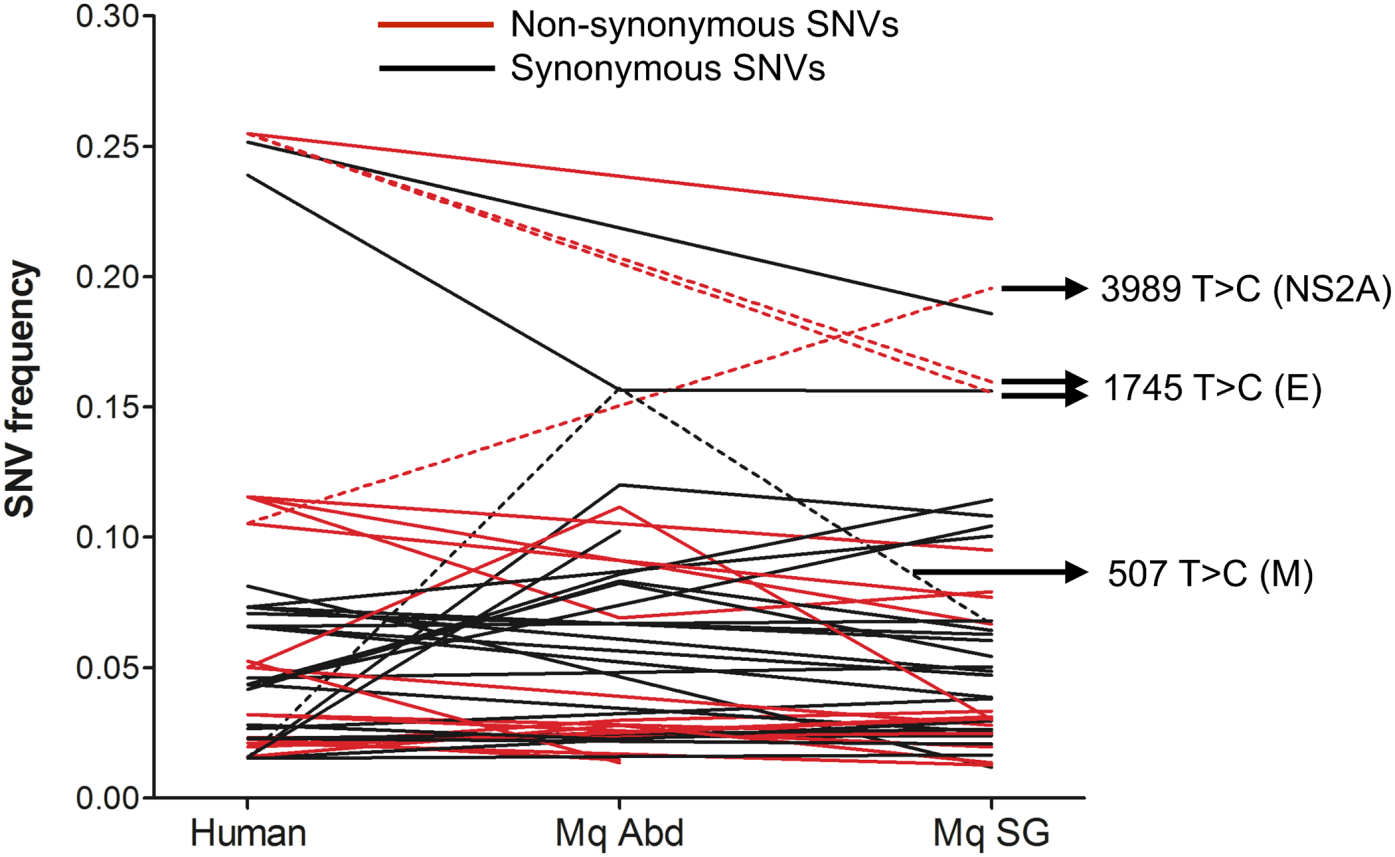
Frequencies of maintained SNVs in the human, mosquito abdomen, and mosquito salivary gland. Red, non-synonymous SNVs; black, synonymous SNVs. SNV positions mentioned in the text are highlighted with arrows and drawn as dotted lines for ease of viewing.

SNVs may also be maintained because they play roles in increasing virus fitness, and selection for these SNVs may result in an increase in their frequencies over the course of transmission. We did not, however, observe this—the frequencies of most maintained SNVs (both synonymous and non-synonymous) remained similar in human-, mosquito abdomen-, and mosquito salivary gland-derived DENV populations (Fig 4), suggesting that most SNVs are neutral in the mosquito. There were several exceptions—for example, the frequency of 3989 T>C (NS2A) nearly doubles from 0.11 in the human to 0.20 in the mosquito, the frequency of 1745 T>C (E) nearly halves from 0.26 in the human to 0.16 in two separate mosquitoes, and the frequency of 507 T>C (M) increases 8-fold from 0.02 in the human to 0.16 in the mosquito abdomen, but drops back to 0.07 in the salivary gland (Fig 4). It is possible that these more dramatic changes in SNV frequency may be due to selection for fitter variants, but it is difficult to differentiate this from stochasticity resulting from drops in population size.

### Paired human-human DENV clinical samples also show predominantly different SNV repertoires

In the EDEN human-human transmission pairs, 15 of 130 SNVs (11.5%) present in the first member of the pair were observed again in the second member (Fig 2C and Table 2). The majority of these SNVs were from a single pair, where 12 out of 23 SNVs (52.2%) in the first member were maintained (S2 Table). Thus, similar to what we observed for human-mosquito pairs, linked human-human pairs of DENV clinical samples also yielded predominantly different SNV repertoires, although there may be exceptions, as observed here. While the majority of SNVs may drop below our detection limit during passage through the mosquito, it is possible that, once transmitted back into the human, selection pressures there may again drive up the frequencies of SNVs that impact virus fitness.

**Table 2:** SNVs maintained between EDEN transmission pairs.

Position	SNV	# Pairs	Gene	NS or S	From (aa)	To (aa)
848	C>T	1	prM	NS	L	F
2566	G>T	1	NS1	NS	G	C
2576	G>T	1	NS1	NS	G	C
2719	G>A	1	NS1	S	G	G
2950	A>G	1	NS1	S	Q	Q
3415	A>T	1	NS1	S	G	G
4060	G>A	1	NS2A	S	L	L
4111	T>C	1	NS2A	S	N	N
4168	A>T	1	NS2B	S	I	I
4441	A>G	1	NS2B	S	V	V
4858	A>G	1	NS3	S	P	P
4873	T>C	1	NS3	S	T	T
4987	A>G	1	NS3	S	T	T
5002	T>C	1	NS3	S	Y	Y
5023	C>T	1	NS3	S	A	A

NS, non-synonymous; S, synonymous; aa, amino acid.

### Estimating virus population drops during transmission

Given that ~90% of SNVs detected in the human were lost, presumably due to chance, during transmission to the mosquito, we wanted to estimate the size of the population drop at which this would occur. For an assumed range of plasma viremia levels from 10^2^ to 10^7^ infectious virions / ml, we estimated the maximum size of the virus population infecting the mosquito for which loss of SNVs could be attributed to random chance. This was done by simulating 1000 samplings of varying sizes from the viral population in the human.

For SNVs with frequencies from 0.01 to 0.03 (the vast majority of SNVs in our dataset), the maximum infecting virus population size for SNV loss to be attributable to chance was 100 virions (S3 Table). This represents the number of virions that establish a productive infection in the mosquito midgut. Since a 2 μl bloodmeal [22,23] taken from a patient with plasma viremia equivalent to the MID50 of 2x10^6^ RNA copies / ml [15] would be expected to contain 4000 RNA copies, this estimate suggests that the viral population size actually infecting the mosquito midgut is reduced at least 40X, probably due to the bulk of viral genomes being non-infectious.

### Selection pressures vary between human and mosquito hosts

Low frequency DENV variants that arise during virus replication are subject to immune selection pressure. To examine differences in selection pressures between hosts, we compared the ratio of the number of non-synonymous to synonymous SNVs (#NS/#S) for each gene in human- versus mosquito-derived virus populations. Because of the small numbers of human plasma-derived DENV2 SNVs from this study, we pooled these with SNVs detected in DENV1 and DENV3 populations from the EDEN study plasma samples [19]. We are aware that selection pressures may differ across DENV serotypes, but this approach allowed us to broadly compare the vertebrate and invertebrate hosts.

**Fig 5:**
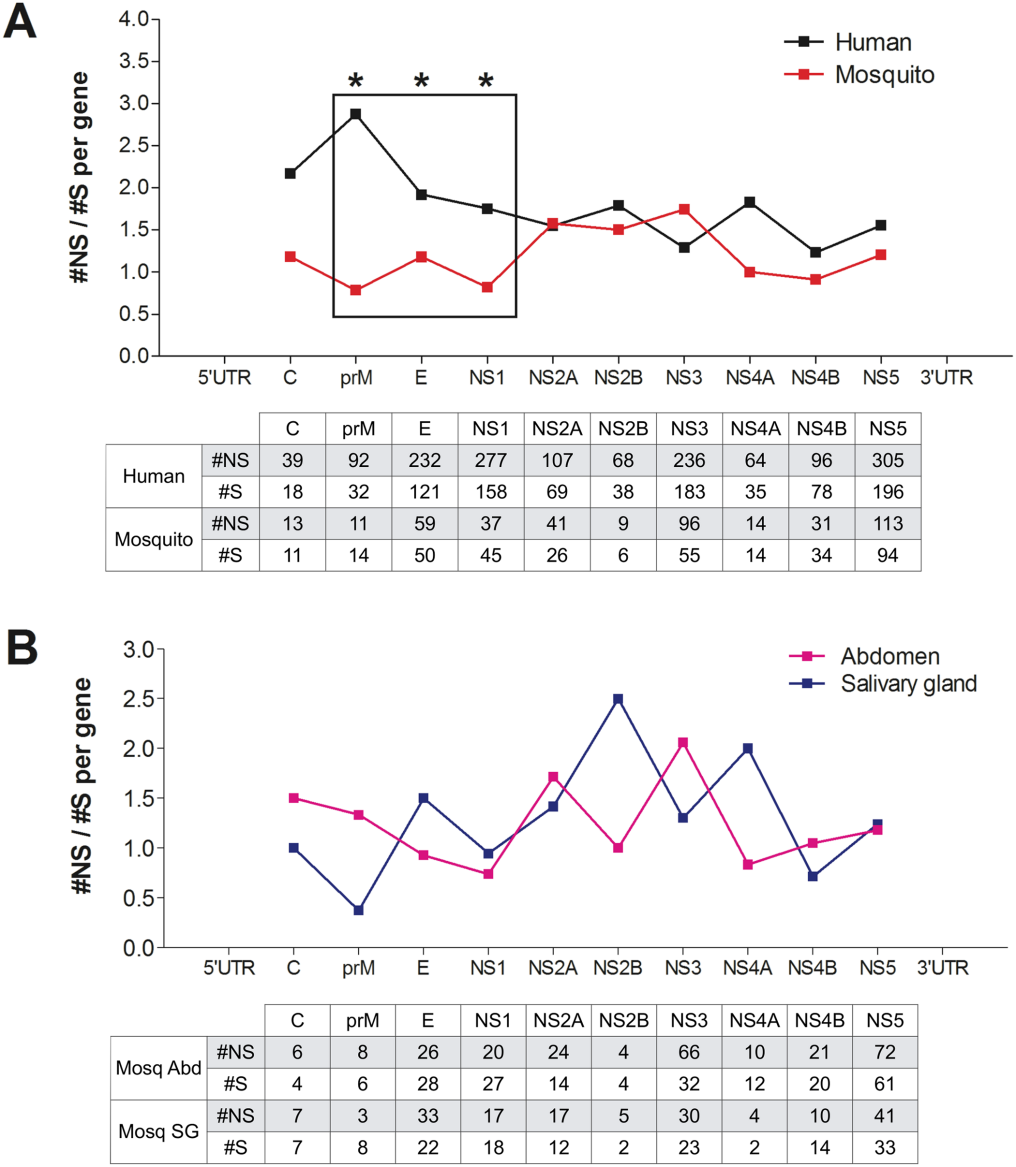
Selection pressures on the DENV genome, analyzed per gene, at different stages of horizontal transmission. Ratios of the number of non-synonymous (NS) to synonymous (S) SNVs per gene are shown for **(A)** human- versus mosquito-derived (both abdomen and salivary gland) DENV populations, and **(B)** mosquito abdomen- versus salivary gland-derived DENV populations. Human-derived samples are a pool of DENV2 patient samples from this study and DENV1 and DENV3 patient samples from the EDEN study [19]. Numbers of NS and S SNVs for each gene are indicated in tables below the graphs. *, p < 0.05; Fisher's exact test (M, p = 0.00439; E, p = 0.031; NS1, p = 0.002).

Human #NS/#S ratios were significantly higher than mosquito ratios for the prM, E, and NS1 genes (Fig 5A). While non-significant, the ratio for C in the human was nearly double that in the mosquito (2.17 versus 1.18). It is striking that the products of these genes are actively targeted by the human antibody response (reviewed in [24]), while no antibody response exists in the mosquito immune repertoire. Although these ratios did not reach the levels formally required for positive selection, we note that positive selection is usually measured at consensus level over time scales much longer than a single transmission [25]. We are instead interested in selection pressures acting on low frequency variants generated over multiple virus replication cycles in each host.

We found no significant differences in #NS/#S ratios between mosquito abdomen and salivary gland for any of the viral genes (Fig 5B), suggesting that host might be more important than body compartment. The much smaller number of SNVs in these two datasets, however, makes it difficult to rule out distinct selection pressures acting in separate mosquito tissues.

**Fig 6:**
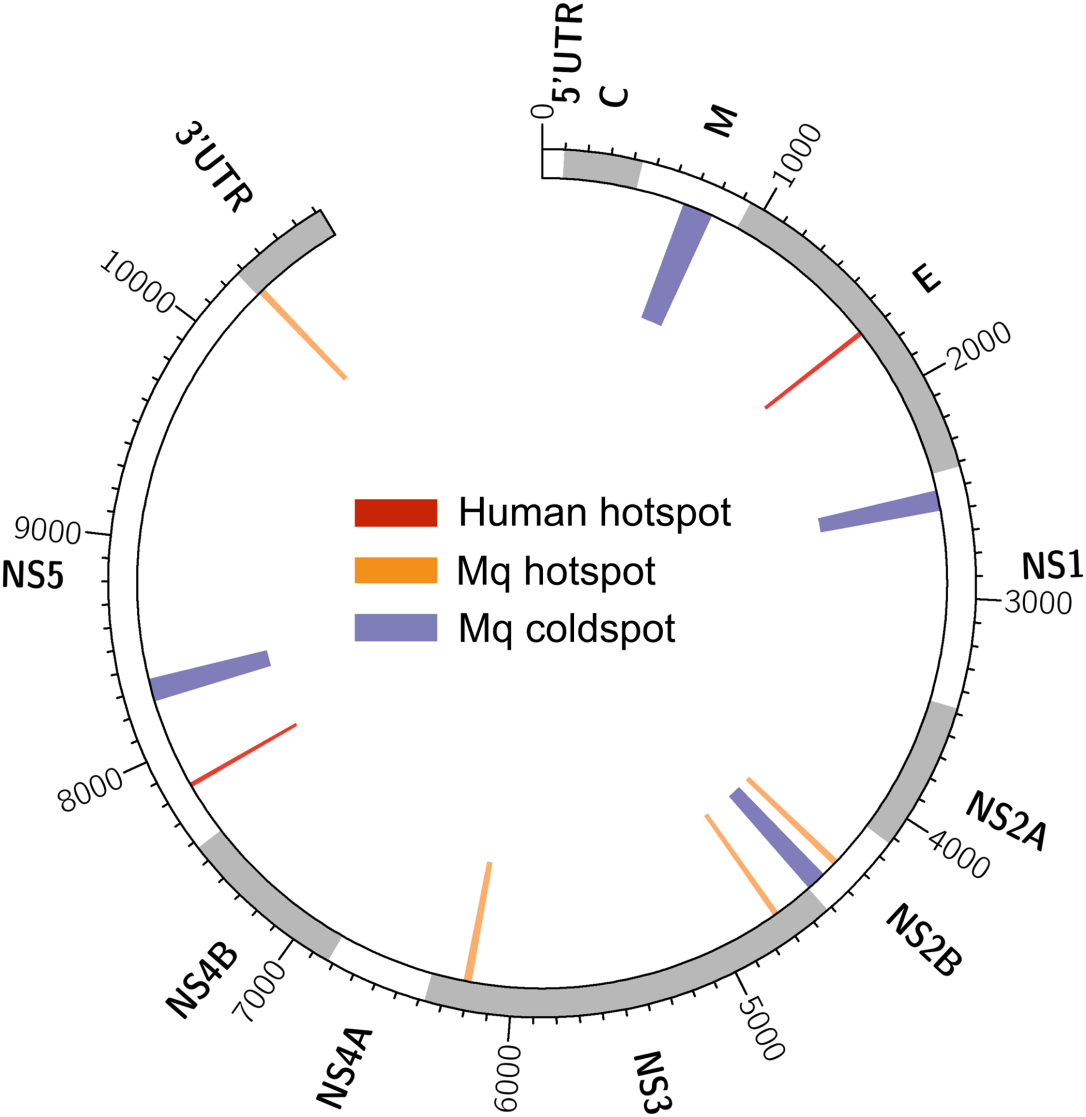
Mutational hot- and coldspots in the DENV2 genome. Circos plot [26] of mutational hot- and coldspots detected in human- and mosquito-derived DENV2 populations. Red, hotspots in human-derived populations; orange, hotspots in mosquito-derived populations; each hotspot was found in a single population. Purple, coldspots indicating a depletion of SNVs across all 46 mosquito-derived populations. No coldspots were detected in human-derived populations.

We also used a scanning window approach to identify mutational hot- and coldspots in the DENV genome; i.e. regions containing a statistically significant excess or lack of SNVs compared with the genome-wide average (Fig 6). We identified a hotspot in E, the gene encoding the DENV envelope protein, in a single human-derived sample from Vietnam, but not in any of the mosquito-derived samples (Fig 6). An E hotspot was also found in a DENV3 human-derived sample from the EDEN study (S4B Fig). These hotspots were located in E domains (ED) II and I respectively, which are known targets of the antibody response [24]. Taken together with our finding that the E #NS/#S ratio is significantly higher in humans compared to mosquitoes (Fig 5A), we speculate that immune pressure on the E protein may play a bigger role in generating diversity in the DENV population in humans than in mosquitoes.

Hotspots in NS3, which encodes the viral serine protease and helicase, were detected in two mosquito-derived samples. While not significantly different between humans and mosquitoes (Fig 5A), the NS3 #NS/#S ratio in mosquitoes was significantly higher than the average across all viral genes (p = 0.027, exact binomial test), suggesting that NS3 may experience a stronger selection pressure than other viral genes in the mosquito. We also identified a 3'UTR hotspot in one mosquito-derived sample. Adaptation to mosquitoes has been proposed as the major driver of evolution in the 3'UTR of chikungunya virus (CHIKV) [27], an alphavirus of the family *Togaviridae*; it will be intriguing to test this hypothesis in DENV, a flavivirus.

The coldspot in prM detected in mosquito-derived samples corresponds to a conserved region of the gene, which has been reported to be important for the association of prM with E during viral assembly [18,28]. We also detected a coldspot in NS5 in mosquito-derived samples, which spans the junction between the methyltransferase and polymerase domains of the protein. The lack of SNVs in these regions suggests the presence of functionally important residues.

27 SNVs (2.4% of all SNVs) were found to encode low frequency premature stop codon mutations. These were spread across the coding region of the DENV genome in both human- and mosquito-derived DENV populations (S4 Table). Previous studies have proposed that defective RNA viruses can be transmitted through complementation by co-infection of host cells with functional virus [29,30]; however in our dataset we did not observe transmission of any of these SNVs.

### Phasing SNVs into distinct variant viral genomes

**Fig 7:**
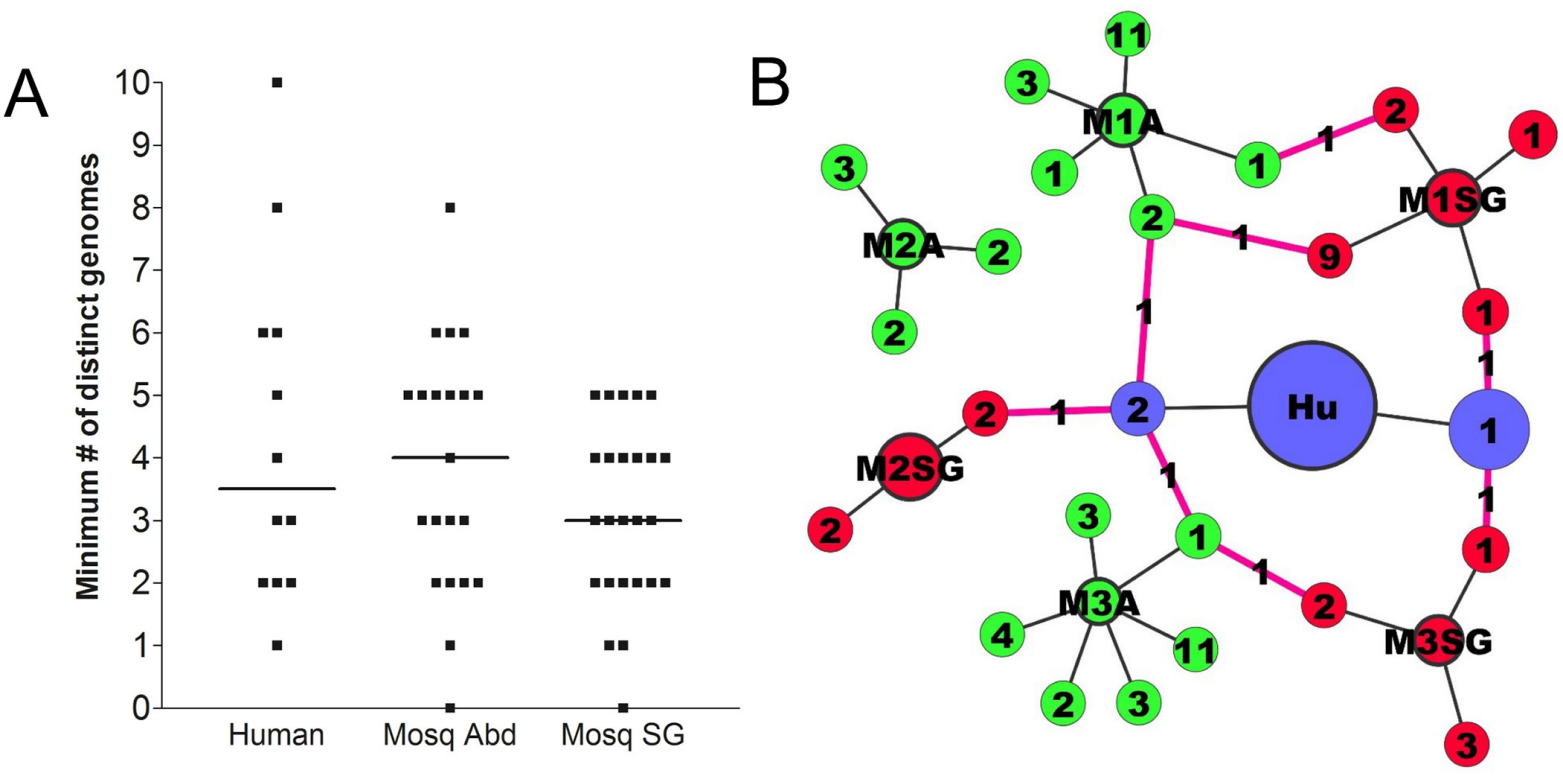
Phasing SNVs into distinct variant viral genomes. **(A)** Minimum numbers of distinct variant DENV genomes per sample, in human- mosquito abdomen-, and mosquito salivary gland-derived DENV2 populations. **(B)** Transmission network for patient-mosquito group 641. For each sample, consensus viral genomes are represented by circles outlined in bold (Hu, human; M1A, M2A, M3A, mosquito abdomens; M1SG, M2SG, M3SG, mosquito salivary glands), and variant genomes by circles radiating out of the consensus circle. Numbers within variant circles indicate the number of SNVs present in each variant genome. Pink connector lines represent maintenance of SNV across transmission stages, with numbers indicating the number of SNVs that were maintained.

To determine how SNVs were distributed into distinct viral genomes, we clustered SNVs in each sample based on frequency, reasoning that SNVs present on the same viral genome would be present at similar frequencies. This gave us an estimate of the minimum number of distinct variant viral genomes in each sample (Fig 7A); the actual number is likely to be higher in many samples, since different genomes could be present at the same frequency, and lower frequency SNVs tend to cluster together without good separation. Similar to the diversity measures in Fig 1, we found no significant difference in the minimum number of distinct variant genomes between virus populations derived from humans, mosquito abdomens, and mosquito salivary glands (p > 0.05, Kruskal-Wallis test) (Fig 7A).

We next used network diagrams to visualize transmission patterns of viral genomes (Fig 7B). In this example (patient-mosquito group 641), no consensus changes were observed as the virus was transmitted from human to mosquito abdomen to mosquito salivary gland. A maximum of only one SNV from each variant genome (shown as circles radiating out of the consensus sequence) was maintained across transmission stages (Fig 7B). This situation was similar across all our other patient-mosquito groups, where it was most common for only one SNV per variant genome to be maintained across transmission stages, with a maximum of two SNVs being maintained as a unit. This suggests that SNVs in each sample were spread out across many variant genomes instead of being clustered together on one genome, and that the actual number of distinct genomes per sample is higher than our predicted minimum number.

## Discussion

Here we report the use of next-generation, whole-genome DENV sequencing to follow within- and between-host differences in viral populations in naturally-infected humans and their infected *Ae*. *aegypti* mosquito counterparts.

We observed that the vast majority (>90%) of SNVs in a population were lost upon virus transmission from human to mosquito. Based on this, we estimated that a maximum of ~100 virions infect the mosquito midgut, with the actual number varying with the level of viremia in the human. This number is consistent with previous studies that used fluorescent reporter viruses to show that, even with high titer bloodmeals, oral infection with WNV or Venezuelan equine encephalitis virus (VEEV, family *Togaviridae*) resulted in a relatively small number of midgut cells being infected, on the order of 15 for WNV [31] and 100 for VEEV [32]. The VEEV study detected a higher than expected frequency of dually-infected cells, suggesting that only a small percentage of midgut cells are susceptible to infection [32].

A 2 μl bloodmeal from a human host where the DENV plasma viremia is 2x10^6^ RNA copies per ml [15] would be expected to contain 4000 RNA copies. Our estimate suggests that the number of virions that actually infect the mosquito midgut is at least 40X lower. This drop, compatible with a bottleneck scenario, could be due to a number of factors, such as the presence of non-infectious particles, preference for certain cell types, and interference with receptor binding, perhaps by the midgut microbiota or proteins in the bloodmeal. For example, the particle-to-PFU (plaque-forming unit) ratio for DENV has been reported to be on the order of 10^3^ to 10^4^: 1 [33,34]. High particle-to-PFU ratios are typical for animal viruses [35], and suggest the presence of many non-infectious particles in a population.

Studies on the effect of population drops on arboviral intra-host genetic diversity have yielded varying results. WNV diversity has been reported to decrease during dissemination from the midgut of *Culex pipiens* to the salivary gland and saliva [36]. Another study reported that WNV genetic diversity was maintained during infection in the mosquito, and, unlike us, observed considerable maintenance of particular variants (cloned sequences) from one mosquito body compartment to another. The authors concluded that in the mosquito, population drops did not significantly impact WNV genetic diversity, and that the maintenance of diversity was more likely due to variation in the input virus population rather than the generation of new mutants [37].

In contrast, intra-host diversity in our DENV dataset was maintained through the generation of a new SNV repertoire at each transmission stage, such that population drops in the mosquito abdomen and salivary glands impact viral repertoire but not overall diversity levels. The high error rate (10^−4^ [4], approximately one mutation per 11 kb DENV genome) and burst size (~10^3^−10^4^ genomes per cell [38,39]) of the virus means that a new array of SNVs is quickly regenerated. It will be interesting to determine if genetic diversity is also maintained in virus populations in mosquito saliva, which this study did not sequence. Infectious DENV loads (PFUs) in saliva vary greatly from mosquito to mosquito, but are in general much lower (~10-100X) than in the salivary gland or carcass [40,41], suggesting that the population injected into a human will have gone through yet another drop. However, our observation that genetic diversity also remains similar across members of EDEN human-human transmission pairs suggests that diversity is quickly restored upon replication in the human.

We observed that SNVs that were maintained between transmission stages were present at significantly higher frequencies than SNVs that were not maintained. A previous next-generation sequencing analysis of human DENV clinical samples from Nicaragua found that intra-host variants that were also observed at the inter-host level (i.e. in consensus sequences of circulating Nicaraguan DENV strains) were present at higher frequencies than variants that were only observed at the intra-host level [7]. This suggests that the initial abundance of a SNV may affect its chances of being maintained and eventually becoming the consensus base. This initial abundance may be subject to stochastic processes; indeed, non-synchronous infection, where early infecting virions have an advantage, has been reported to be a major contributor towards genetic drift in human immunodeficiency virus (HIV) populations [42].

We were unable to follow transmission from the mosquito back into the human, but the EDEN human-human transmission pairs (thought to be separated by a single mosquito) allowed us to make hypotheses about this process. Similar to the human-mosquito pairs, paired human-human DENV clinical samples also showed predominantly different SNV repertoires. We estimate that the majority of SNVs present in a human sample will fall below our detection limit during passage through the mosquito, where most SNVs appear to be neutral. However, once transmitted to a second human host, selection pressures there may once again drive up the frequencies of SNVs that impact virus fitness in the human. Our observation that several viral genes display higher #NS/#S ratios in human- than in mosquito-derived samples is consistent with this idea, but SNVs would have to be followed over a chain of multiple linked transmissions in order to further investigate this.

Over multiple rounds of replication in the human or mosquito, viral populations grow exponentially and generate a range of low frequency variants that are subject to selection pressures. Our data suggest that variants in the prM, E, and NS1 genes experience stronger immune pressures in the human than in the mosquito, consistent with the fact that these gene products are known targets of the human antibody response (reviewed in [24]), which has no equivalent in mosquitoes. The envelope protein E is the main antigen on the virion surface and the target of neutralizing antibody [43,44]. Intra-host genetic diversity in the various E protein domains has been reported to positively correlate with immunogenicity in humans, suggesting that immune-driven selection occurs even during short-term, acute dengue infection [7]. We further observed E mutational hotspots in human- but not mosquito-derived DENV populations, leading us to hypothesize that the antibody response in humans is a major driver of viral diversity.

The mosquito immune response lacks an adaptive arm, but is able to mount potent responses against virus, bacteria, and fungi through the Toll, IMD, and JAK/STAT immune signaling pathways (reviewed in [45]). Compared to the situation in vertebrates, relatively little is known about the molecular mechanisms by which these pathways act against DENV, and a better understanding is required before selection pressures on DENV in the vector can be characterized.

In addition to the classical immune signaling pathways, RNAi is also a major antiviral defense mechanism in the mosquito [40,46]. WNV intra-host genetic diversity is thought to be driven by RNAi, with the parts of the WNV genome most likely to be targeted by RNAi also being the most diverse [8]. A separate study made use of artificially diverse WNV strains to show that high intra-host genetic diversity was associated with increased viral fitness in *Culex* mosquitoes [47]. Although it has been proposed that purifying selection is relaxed in the *Culex* mosquito host for WNV [10,48], these studies suggest that diversity may be actively selected for in insect vectors. This idea is supported by our observation that almost entirely new DENV SNV repertoires were generated at each transmission stage, but requires further testing in the DENV-*Ae*. *aegypti* system.

We found no evidence for differences in selection pressures between the mosquito abdomen and salivary gland. Still, because the SNV sample size was small, this does not rule out differences in immune pressures between body compartments, and #NS/#S ratios did diverge for several genes. A potential driver of differing immune pressures is the midgut microbiota, which has been shown to impact mosquito physiology and vector competence for human pathogens (reviewed in [49]). Depletion of the gut microbiota increases mosquito susceptibility to DENV [50]; this is thought to occur at least partly because the microbiota trigger a basal level of immune activity [50–52]. It will be interesting to examine the impact of the gut microbiota on DENV diversity.

After phasing SNVs into distinct viral genomes, we observed that in most cases, only one SNV from a predicted variant genome was maintained across transmission stages, with a maximum of two being maintained as a unit. This suggests that SNVs in a sample are spread out across many variant genomes instead of being clustered together on one genome, and is consistent with the RdRp error rate of 1x10^-4^ [4], which corresponds to approximately one mutation per 11 kb DENV genome.

Despite the growing number of studies characterizing intra-host genetic diversity, the impact of this diversity on virulence or disease severity for arboviruses in vertebrate hosts is not well understood. One study unexpectedly found that mouse morbidity and mortality was negatively correlated with WNV intra-host genetic diversity [10]. The authors suggest that this could be due to the parental virus clone being highly pathogenic, such that most mutations would result in a decrease in pathogenicity. Alternatively, adaptation to mosquitoes during passage could also have resulted in a loss of pathogenicity [10]. Another study found lower intra-host diversity in patients with severe dengue than with mild dengue [12]; however the authors state that it is difficult to know if these differences are the cause or the consequence of disease severity. In other studies, no association was found between DENV intra-host diversity and disease severity [7,14]. It will be important to address the question of whether disease severity is associated with the genetic diversity of the infecting viral population, rather than with diversity after the onset of symptoms.

## Supporting Information

S1 Fig Dose–response scatterplots of the proportion of DENV2-infected mosquitoes after blood-feeding on 12 dengue patients, versus the plasma viremia of these patients. (TIF)

S2 Fig Distribution of SNVs across the DENV2 genome. 1116 SNVs and their frequencies are displayed here.
(TIF)

S3 Fig Diversity measures for clinical DENV samples from the Early DENgue (EDEN) study [19]. (A) Number of SNVs; (B) Sum of SNV frequencies; (C) Average SNV frequency; (D) Standard error of the mean SNV frequency; all calculated on a per sample basis.
(TIF)

S4 Fig Mutational hot- and coldspots in EDEN clinical DENV samples. Circos plots [26] of mutational hot and coldspots detected in **(A)** DENV1 and **(B)** DENV3 clinical samples from the EDEN study. Red, hotspots; each hotspot was found in a single sample; blue, coldspots, indicating a depletion of SNVs across 33 DENV1 and 27 DENV3 samples.
(TIF)

S1 Table Characteristics of 12 dengue cases that were exposed to *Ae*. *aegypti* mosquitoes, and successfully sequenced mosquito samples. DOI, day of illness at time of mosquito exposure; Fever day, day (at the time of mosquito exposure) compared to the defervescence day, which is defined as fever day 0. Defervescence day is the day when patient's temperature returns to less than 37.5°C and remains below this threshold until discharge. One day before defervescence day is fever day -1; one day after defervescence day is fever day +1.
(DOCX)

S2 Table EDEN transmission pairs. Human-human pairs of EDEN clinical DENV samples predicted to be separated by one mosquito are shown, along with the degree of SNV overlap between members of a pair.
(DOCX)

S3 Table Estimating population drops during transmission. The maximum infecting virus population size for which SNV loss could be attributable to chance (p > 0.05) was calculated by simulating 1000 samplings of varying size from the virus population in the human, which was assumed to range from 2x10^2^ to 2x10^7^ infectious virions / ml or 1x10^6^ to 1x10^11^ in 5 liters of blood.
(XLSX)

S4 Table SNVs encoding premature stop codon mutations. (DOCX)

S5 Table Sequences of primers used to amplify the DENV2 genome in 14 overlapping fragments. (DOCX)
