# Supplementary figures and images for: Tracking Dengue Virus Intra-host Genetic Diversity during Human-to-Mosquito Transmission
Source: PLoS Negl Trop Dis. 2015 Sep 1;9(9):e0004052. doi: 10.1371/journal.pntd.0004052 (PMC4556672; doi:10.1371/journal.pntd.0004052)

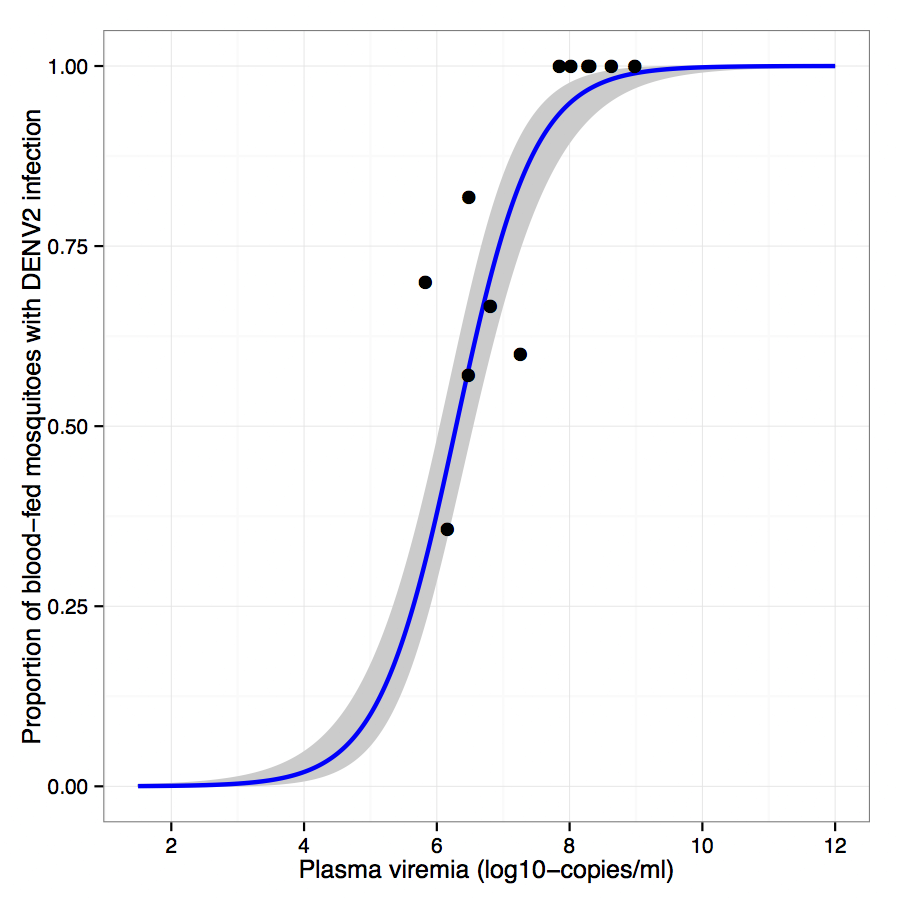

Supplement: S1 Fig — (TIF) [file pntd.0004052.s001.tif]

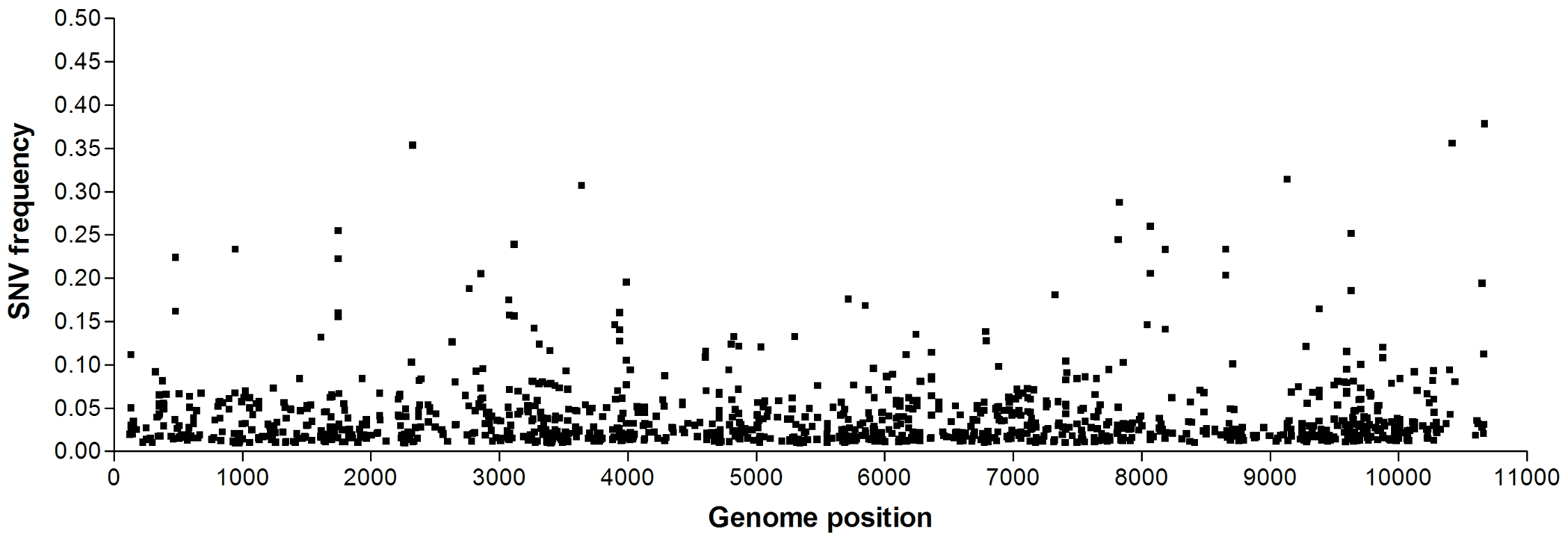

Supplement: S2 Fig — 1116 SNVs and their frequencies are displayed here. (TIF) [file pntd.0004052.s002.tif]

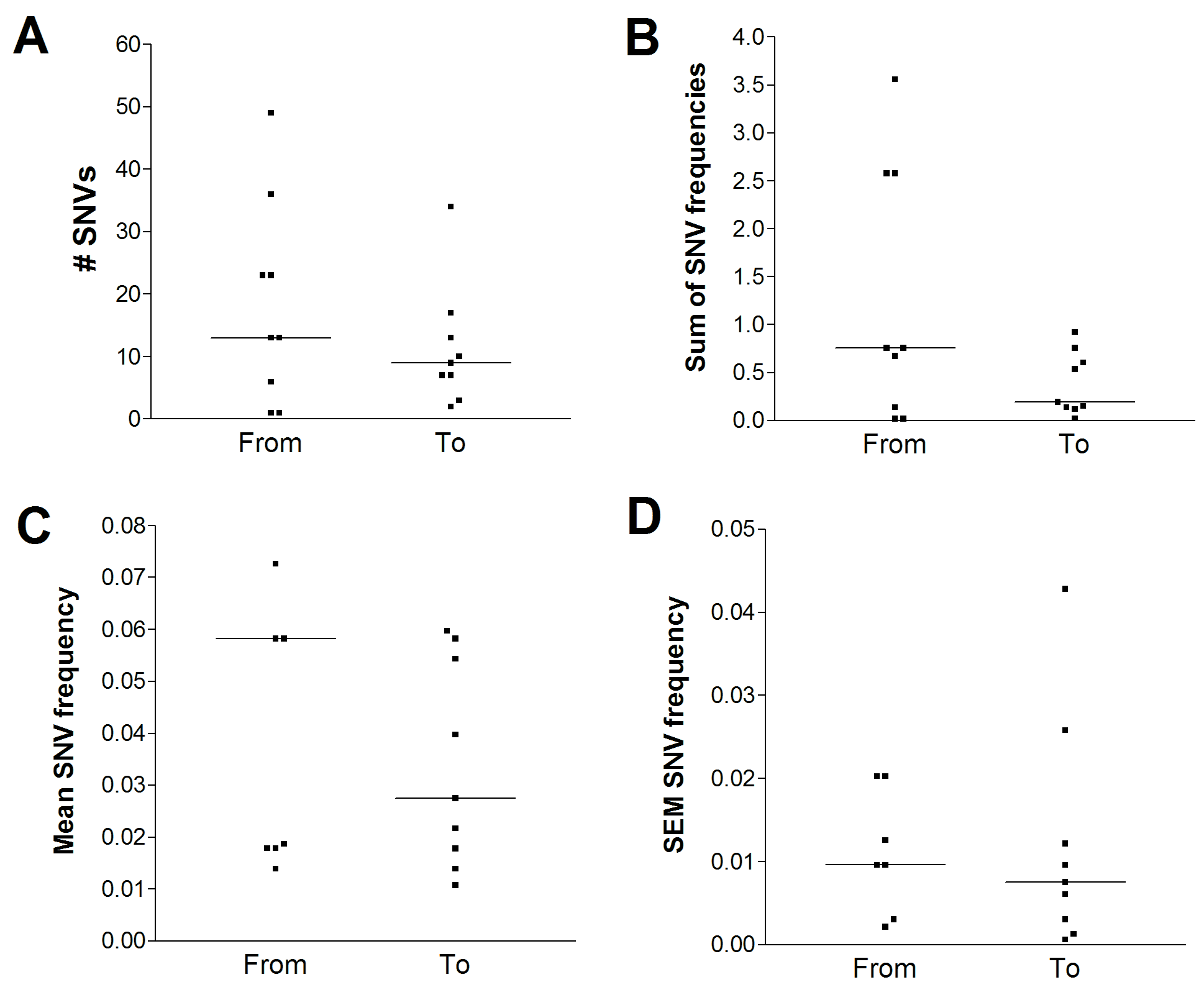

Supplement: S3 Fig — (A) Number of SNVs; (B) Sum of SNV frequencies; (C) Average SNV frequency; (D) Standard error of the mean SNV frequency; all calculated on a per sample basis. (TIF) [file pntd.0004052.s003.tif]

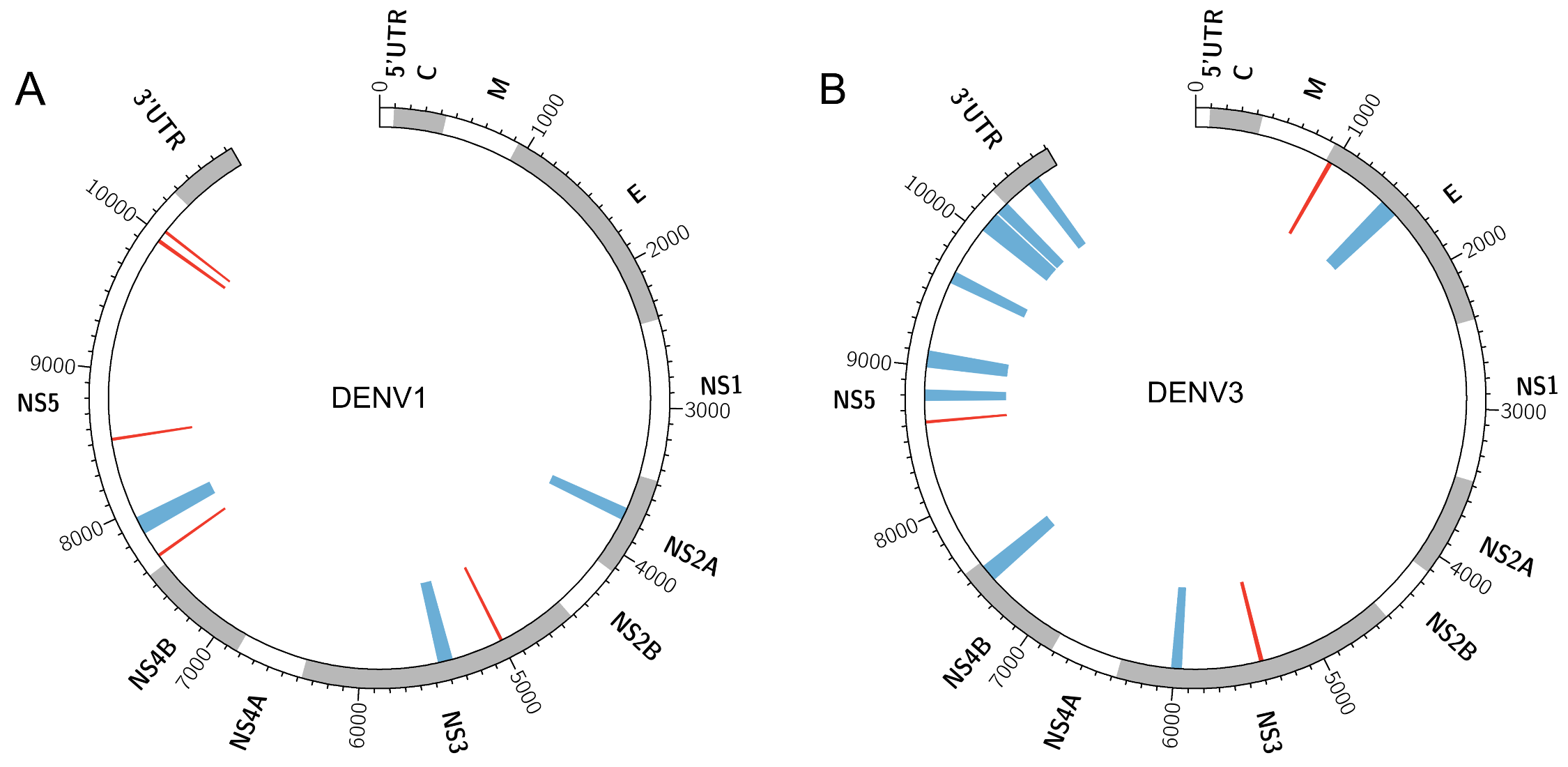

Supplement: S4 Fig — Circos plots [26] of mutational hot and coldspots detected in (A) DENV1 and (B) DENV3 clinical samples from the EDEN study. Red, hotspots; each hotspot was found in a single sample; blue, coldspots, indicating a depletion of SNVs across 33 DENV1 and 27 DENV3 samples. (TIF) [file pntd.0004052.s004.tif]
