# Supplementary material for: Tracking Dengue Virus Intra-host Genetic Diversity during Human-to-Mosquito Transmission
Source: PLoS Negl Trop Dis. 2015 Sep 1;9(9):e0004052. doi: 10.1371/journal.pntd.0004052 (PMC4556672; doi:10.1371/journal.pntd.0004052)
Supplement: S1 Table — DOI, day of illness at time of mosquito exposure; Fever day, day (at the time of mosquito exposure) compared to the defervescence day, which is defined as fever day 0. Defervescence day is the day when patient's temperature returns to less than 37.5°C and remains below this threshold until discharge. One day before defervescence day is fever day -1; one day after defervescence day is fever day +1. (DOCX) [file pntd.0004052.s005.docx]

**Table S1: Characteristics of 12 dengue cases that were exposed to *Ae. aegypti* mosquitoes, and successfully sequenced mosquito samples.** DOI, day of illness at time of mosquito exposure; Fever day, day (at the time of mosquito exposure) compared to the defervescence day, which is defined as fever day 0. Defervescence day is the day when patient's temperature returns to less than 37.5°C and remains below this threshold until discharge. One day before defervescence day is fever day -1; one day after defervescence day is fever day +1.

| **Study No.** | **Plasma sample** | **DOI** | **Fever day** | **Serotype** | **Patient age** | **Patient sex** | **Enrolment viremia (log10 copies/ml)** | **Exposure viremia (log10 copies/ml)** | **Exposure temperature (°C)** | **Proportion of infected mosquitoes** | **Mosquito abdomen sample** | | | **Mosquito salivary gland sample** | | |
| --- | --- | --- | --- | --- | --- | --- | --- | --- | --- | --- | --- | --- | --- | --- | --- | --- |
| 613 | 613 - 102 | 4 | -2 | DENV2 | 20 | Female | 6.606 | 6.475 | 39.7 | 0.571 |  |  |  |  |  |  |
| 620 | 620 - 102 | 4 | -2 | DENV2 | 23 | Female | 7.636 | 8.305 | 38.2 | 1.000 |  |  |  | 620-102-M1 | 620-102-M2 | 620-102-M3 |
| 626 | 626 - 103 | 5 | 1 | DENV2 | 22 | Female | 7.297 | 6.808 | 37.1 | 0.667 |  |  |  |  |  |  |
| 629 | 629 - 102 | 4 | -2 | DENV2 | 31 | Female | 8.800 | 7.850 | 38.6 | 1.000 | 629-102-M1 | 629-102-M2 | 629-102-M3 | 629-102-M1 | 629-102-M2 | 629-102-M3 |
| 641 | 641 - 102 | 3 | -5 | DENV2 | 28 | Female | 9.285 | 8.984 | 37.9 | 1.000 | 641-102-M1 | 641-102-M2 | 641-102-M3 | 641-102-M1 | 641-102-M2 | 641-102-M3 |
| 643 | 643 - 103 | 5 | 0 | DENV2 | 30 | Female | 8.107 | 5.832 | 36.8 | 0.700 |  |  |  | 643-103-M1 |  |  |
| 652 | 652 - 101 | 3 | -4 | DENV2 | 24 | Female | 8.279 | 8.279 | 37.1 | 1.000 |  |  |  |  |  |  |
| 809 | 809 - 103 | 5 | 1 | DENV2 | 31 | Male | 7.892 | 6.486 | 36.8 | 0.818 | 809-103-M1 | 809-103-M2 | 809-103-M3 | 809-103-M1 | 809-103-M2 | 809-103-M3 |
| 816 | 816 - 102 | 4 | -1 | DENV2 | 39 | Male | 4.828 | 6.164 | 37 | 0.357 | 816-102-M1 | 816-102-M2 | 816-102-M8 | 816-102-M1 | 816-102-M2 | 816-102-M8 |
| 827 | 827 - 101 | 2 | -2 | DENV2 | 17 | Male | 8.021 | 8.021 | 37.9 | 1.000 | 827-101-M1 | 827-101-M2 | 827-101-M3 | 827-101-M1 | 827-101-M2 | 827-101-M3 |
| 841 | 841 - 102 | 4 | -3 | DENV2 | 23 | Female | 9.146 | 8.634 | 37.1 | 1.000 | 841-102-M1 | 841-102-M2 | 841-102-M3 | 841-102-M1 | 841-102-M2 | 841-102-M3 |
| 847 | 847 - 102 | 4 | -1 | DENV2 | 27 | Male | 7.633 | 7.262 | 36.8 | 0.600 | 847-102-M1 | 847-102-M2 | 847-102-M4 | 847-102-M1 | 847-102-M2 | 847-102-M4 |
