# Supplementary material for: Tracking Dengue Virus Intra-host Genetic Diversity during Human-to-Mosquito Transmission
Source: PLoS Negl Trop Dis. 2015 Sep 1;9(9):e0004052. doi: 10.1371/journal.pntd.0004052 (PMC4556672; doi:10.1371/journal.pntd.0004052)
Supplement: S2 Table — Human-human pairs of EDEN clinical DENV samples predicted to be separated by one mosquito are shown, along with the degree of SNV overlap between members of a pair. (DOCX) [file pntd.0004052.s006.docx]

**Table S2: EDEN transmission pairs.** Human-human pairs of EDEN DENV isolates predicted to be separated by one mosquito are shown, along with the degree of SNV overlap between members of a pair.

| **Pair** | **From** | **To** | **# days apart** | **Serotype** | **Physical distance (metres)** | **# SNVs (From)** | **# SNVs (To)** | **# common SNVs** | **% maintained (of first member of pair)** |
| --- | --- | --- | --- | --- | --- | --- | --- | --- | --- |
| 1 | 2398 | 2887 | 9 | DENV1 | >500 | 23 | 17 | 12 | 52.2 |
| 2 | 2398 | 2901 | 15 | DENV1 | >500 | 23 | 10 | 0 | 0.0 |
| 3 | 2901 | 3297 | 20 | DENV1 | >500 | 6 | 13 | 1 | 16.7 |
| 4 | 3297 | 3903 | 16 | DENV1 | >500 | 13 | 3 | 0 | 0.0 |
| 5 | 3297 | 3904 | 16 | DENV1 | >500 | 13 | 7 | 0 | 0.0 |
| 6 | 3894 | 3915 | 5 | DENV1 | >500 | 36 | 9 | 1 | 2.8 |
| 7 | 827 | 863 | 19 | DENV3 | 150-500 | 49 | 34 | 0 | 0.0 |
| 8 | 843 | 868 | 9 | DENV3 | >500 | 1 | 7 | 1 | 100.0 |
| 9 | 843 | 871 | 10 | DENV3 | >500 | 1 | 2 | 1 | 100.0 |
