# Supplementary material for: Tracking Dengue Virus Intra-host Genetic Diversity during Human-to-Mosquito Transmission
Source: PLoS Negl Trop Dis. 2015 Sep 1;9(9):e0004052. doi: 10.1371/journal.pntd.0004052 (PMC4556672; doi:10.1371/journal.pntd.0004052)
Supplement: S4 Table — (DOCX) [file pntd.0004052.s008.docx]

**Table S4: SNVs encoding premature stop codon mutations.**

| **Isolate ID** | **Type** | **SNV position** | **SNV** | **From (amino acid)** | **To** | **Coverage** | **Frequency** | **Gene** |
| --- | --- | --- | --- | --- | --- | --- | --- | --- |
| 613 | Human | 460 | G>T | G | * | 28794 | 0.014595 | prM |
| 816M2T | Mosquito | 973 | G>T | E | * | 14437 | 0.010028 | E |
| 816M2A | Mosquito | 1212 | C>A | C | * | 36954 | 0.029618 | E |
| 643M1T | Mosquito | 1822 | G>T | G | * | 40290 | 0.038663 | E |
| 827M2A | Mosquito | 2445 | G>A | W | * | 22982 | 0.053783 | NS1 |
| 613 | Human | 2765 | G>A | W | * | 1579 | 0.188113 | NS1 |
| 841 | Human | 2870 | G>A | W | * | 3858 | 0.095674 | NS1 |
| 847M2T | Mosquito | 3225 | G>A | W | * | 10041 | 0.013117 | NS1 |
| 816M3A | Mosquito | 3361 | C>T | R | * | 14208 | 0.02493 | NS1 |
| 816 | Human | 3414 | C>G | Y | * | 8332 | 0.014486 | NS1 |
| 827M1A | Mosquito | 4728 | G>A | W | * | 41520 | 0.011481 | NS3 |
| 841M2A | Mosquito | 5107 | G>T | G | * | 14982 | 0.014683 | NS3 |
| 816M3A | Mosquito | 5552 | G>A | W | * | 20703 | 0.010482 | NS3 |
| 641M1A | Mosquito | 5553 | G>A | W | * | 60183 | 0.011869 | NS3 |
| 643 | Human | 5553 | G>A | W | * | 14674 | 0.032317 | NS3 |
| 629M1T | Mosquito | 5703 | T>G | Y | * | 10036 | 0.011733 | NS3 |
| 841M2A | Mosquito | 6207 | G>A | W | * | 19572 | 0.015838 | NS3 |
| 816M1A | Mosquito | 7179 | T>G | Y | * | 17845 | 0.027714 | NS4B |
| 816M2T | Mosquito | 7607 | G>A | W | * | 6915 | 0.027346 | NS5 |
| 816M2T | Mosquito | 7608 | G>A | W | * | 6935 | 0.027582 | NS5 |
| 620M2T | Mosquito | 8708 | G>A | W | * | 2751 | 0.020578 | NS5 |
| 841M3A | Mosquito | 8709 | G>A | W | * | 4426 | 0.101048 | NS5 |
| 841M1A | Mosquito | 9060 | G>A | W | * | 17637 | 0.015198 | NS5 |
| 841 | Human | 9507 | G>A | W | * | 22739 | 0.01403 | NS5 |
| 816M2T | Mosquito | 9847 | C>T | Q | * | 8147 | 0.035806 | NS5 |
| 827 | Human | 10008 | G>A | W | * | 57642 | 0.029004 | NS5 |
| 841M2A | Mosquito | 10069 | G>T | E | * | 19568 | 0.023615 | NS5 |
