# Supplementary material for: Tracking Dengue Virus Intra-host Genetic Diversity during Human-to-Mosquito Transmission
Source: PLoS Negl Trop Dis. 2015 Sep 1;9(9):e0004052. doi: 10.1371/journal.pntd.0004052 (PMC4556672; doi:10.1371/journal.pntd.0004052)
Supplement: S5 Table — (DOCX) [file pntd.0004052.s009.docx]

**Table S5: Sequences of primers used to amplify the DENV2 genome in 14 overlapping fragments.**

| **Fragment** | **Forward** | **Reverse** | **Region** |
| --- | --- | --- | --- |
| 1 | AGTAGTTAGTCTACGTGGACCG | GATCCRAAATCCCARGCTGTGTC | 1-2207 |
| 2 | CAGATCTCTGATGAATAACCAACG | GATCCRAAATCCCARGCTGTGTC | 87-2207 |
| 3 | CCAGAAGACATAGAYTGTTGGTG | TRCCTGCATGATTCCTTTRATGTC | 619-2718 |
| 4 | TGCCCAACACAAGGRGAACCYA | ATCTTCCATGTRTCATTGAGTGC | 1156-3056 |
| 5 | ATGGTGCARGCYGATAGTGGTT | TAGGCTCCRTCTTCCAGTTCRG | 2410-4589 |
| 6 | YATGACAGGAGACATYAAAGGAATC | CTTCCARCCTCCTCCATAYGATA | 2685-4773 |
| 7 | ATGCTYAGGACCCGAGTAGGAA | AGRCAAGCTGCTATRTCATTTCC | 3541-5645 |
| 8 | GCATGGAARGTGAGYTGCACAA | TATTCRCCRTCAATGGCATCCAC | 3967-6089 |
| 9 | GTYACAAGGAGTGGARCATATGT | AGRAGGGGAACTCCRATGTCCA | 4984-7106 |
| 10 | GCAGCYGGGATTTTYATGACAG | GTYTCTCCTRTGTTGCCAGTTC | 5446-7592 |
| 11 | AGATGGYTGGAYGCTAGGATCTA | TARTGCCATGAYGTTTCATGCTC | 6301-8450 |
| 12 | CTAGAWCCAATACCYTATGATCC | GTGATTCTTGTGTCCCAKCCTG | 7288-9197 |
| 13 | GTGACATAGGGGARTCRTCACC | GTARTCTGTGTATTCCTCATTGCC | 8003-10215 |
| 14 | GCCATATTCACYGATGAGAACAAR | AGAACCTGTTGATTCAACAGCAC | 8800-10723 |
